# Quantitative evaluation of fMRI retinotopic maps, from V1 to V4, for cognitive experiments

**DOI:** 10.3389/fnhum.2015.00277

**Published:** 2015-05-19

**Authors:** Cécile Bordier, Jean-Michel Hupé, Michel Dojat

**Affiliations:** ^1^Grenoble Institut des Neurosciences, Université Grenoble AlpesGrenoble, France; ^2^Inserm, U836Grenoble, France; ^3^Centre de Recherche Cerveau et Cognition, Université de Toulouse and Centre National de la Recherche ScientifiqueToulouse, France

**Keywords:** functional imaging, human vision, MRI, occipital cortex, phase-encoded design, Retinotopy, V4

## Abstract

FMRI retinotopic mapping is a non-invasive technique for the delineation of low-level visual areas in individual subjects. It generally relies upon the analysis of functional responses to periodic visual stimuli that encode eccentricity or polar angle in the visual field. This technique is used in vision research when the precise assignation of brain activation to retinotopic areas is an issue. It involves processing steps computed with different algorithms and embedded in various software suites. Manual intervention may be needed for some steps. Although the diversity of the available processing suites and manual interventions may potentially introduce some differences in the final delineation of visual areas, no documented comparison between maps obtained with different procedures has been reported in the literature. To explore the effect of the processing steps on the quality of the maps obtained, we used two tools, BALC, which relies on a fully automated procedure, and BrainVoyager, where areas are delineated “by hand” on the brain surface. To focus on the mapping procedures specifically, we used the same SPM pipeline for pretreatment and the same tissue segmentation tool. We document the consistency and differences of the fMRI retinotopic maps obtained from “routine retinotopy” experiments on 10 subjects. The maps obtained by skilled users are never fully identical. However, the agreement between the maps, around 80% for low-level areas, is probably sufficient for most applications. Our results also indicate that assigning cognitive activations, following a specific experiment (here, color perception), to individual retinotopic maps is not free of errors. We provide measurements of this error, that may help for the cautious interpretation of cognitive activation projection onto fMRI retinotopic maps. On average, the magnitude of the error is about 20%, with much larger differences in a few subjects. More variability may even be expected with less trained users or using different acquisition parameters and preprocessing chains.

## 1. Introduction

In humans, the visual cortex is organized into different functional areas where adjacent neurons have receptive fields sensitive to adjacent positions in the visual field. Neurons from these areas define a continuous mapping between the visual field and the cortical surface. Based on what has been called the retinotopy property and using functional Magnetic Resonance Imaging (fMRI) we can non-invasively delineate visual cortical areas. Based on the seminal papers of Engel et al. (1994); Sereno et al. (1995); DeYoe et al. (1996), different methodological refinements have been proposed (Slotnick and Yantis, 2003; Kraft et al., 2005; Vanni et al., 2005; Dumoulin and Wandell, 2008; Henriksson et al., 2012) for the robust and reproducible delineation of low-level areas in an individual subject. At the border between adjacent visual areas, the local cortical representation of the visual field is inverted, mapping a direct or a mirror image of the visual field (often called inversion of the “visual field sign”). The orientation of the cortical representation of the visual field alternates between adjacent areas (Sereno et al., 1994). The most popular technique for fMRI retinotopic mapping relies upon analysis of functional responses to periodic visual stimuli (annulus or wedge, both centered on a fixation point) that encode eccentricity or polar angle in the visual field (Engel et al., 1994). These stimuli associate each position in the visual field to a delay of the periodic stimulation of the neurons with the corresponding receptive fields. Although perfusion contrast can provide an interesting alternative (Cavusoglu et al., 2012), this delay is generally measured with BOLD contrast and conveniently expressed in term of phase of the BOLD signal in the frequency domain. Phase analysis can be performed in a 3D-space (Dumoulin et al., 2003) or in the surface to detect more precisely and possibly automatically where the inversion of the visual field sign occurs. This often called phase-encoded functional design (Engel, 2012) is now largely used in vision research, when the precise assignation of functional activation to specific retinotopic areas is an issue. The technique is powerful enough to reveal the retinotopic organization of subcortical structures such as the lateral geniculate nucleus (Schneider et al., 2004) or the superior colliculus (Schneider and Kastner, 2005). Despite some limitations notably in the foveal region (Wandell and Winawer, 2011) or beyond V3v (Winawer et al., 2010), retinotopic maps are measured reliably by phase-encoded designs both in ventral (Wade et al., 2002; Arcaro et al., 2009) and dorsal (Larsson and Heeger, 2006; Pitzalis et al., 2006; Hansen et al., 2007; Amano et al., 2009) streams.

The boundaries between the lower visual areas V1, V2, and V3 are in general not difficult to trace without prior assumptions about the visual field map layout (Dougherty et al., 2003; Wandell et al., 2007). Such visual field maps provide a definition of regions of interest across subjects more robust and consistent than some debatable functional localizers (Friston et al., 2006; Saxe et al., 2006). New advances in MR signal measurement (multi-channel coil Schira et al., 2009) or moving from 3T to 7T magnetic field strength (Hoffmann et al., 2009; Olman et al., 2010) could help to refine the description of retinotopic maps in reducing voxel size (spatial resolution improvement) and effects of the pial veins. This provides better data in the ventral occipital region (Arcaro et al., 2009) or dorsal V3 and beyond (Larsson and Heeger, 2006; Amano et al., 2009). Spatial attention directed to the mapping stimulus instead of the central fixation cross could increase the reliability of the measured responses (Bressler and Silver, 2010). Retinotopic mapping involves specific data acquisition and data processing steps. Successful retinotopic mapping requires a high-quality structural image in which the contrast-to-noise ratio is high enough to allow for a robust segmentation of the white matter - gray matter interface. For surface analysis or proper visualization of the localization of the borders between visual areas, an explicit model of the cortical surface is processed. For this purpose, the structural image is segmented and the cortical surface is reconstructed under the form of a mesh and, for visualization purposes, unfolded or flattened. Removal of topological defects if any can be done automatically (Fischl et al., 2001; Kriegeskorte and Goebel, 2001) and refined manually. Functional activations are assigned to the surface via interpolation to the positions corresponding to the nodes of the cortical mesh. The quality of the alignment between functional and anatomical data is then crucial to avoid error in the surface representation of the functional data. Minimal distortion in the functional data (BOLD contrast, T2^*^-weighted scans) is then a prerequisite to ensure an accurate registration with the structural reference (T1-weighted scan). Depending on the hardware available or acquisition conditions, correction of the spatial distortions due to static field inhomogeneity should be introduced (Vasseur et al., 2010). Slight errors in segmentation or alignment can hamper the cortical organization assessment (Olman et al., 2010). Spatial smoothing in the surface and detection of visual field sign allow manual or automatic delineation for retinotopic visual areas. The visual field sign can be computed as the Jacobian of the visual field representation on the surface (Sereno et al., 1995; Warnking et al., 2002). All these steps rely on various methods embedded in different software suites such as BALC (Warnking et al., 2002), MrVista (Wandell et al., 2000), BrainVoyager (Goebel et al., 2006), FreeSurfer (Fischl et al., 2001), or Caret (Van Essen et al., 2001).

We hypothesized that the diversity of the suites used and the manual intervention needed for some steps (i.e., functional and structural perfect alignment in the occipital lobe, correction of cortical volume topology or delineation of the areas borders) may introduce potential differences on the final visual area localization. The goal of our study was to document these differences if any and to explore their impact on the interpretation of the cognitive activation projected onto these maps. For this purpose, we report the retinotopic maps obtained using two experts and two software suites: BALC and BrainVoyager and document their consistency and differences. Generally, fMRI retinotopic maps are computed and then used as localizers in relation with a specific cognitive experiment. Indeed, cortical activation produced by a cognitive experiment in individuals is projected onto the corresponding retinotopic maps revealing which visual areas are involved in information processing. We document the consistency between color center mapping data projection using one chain or the other. Our goal was not to demonstrate the superiority of one solution to the other. These software were chosen because they were mastered by the authors and were representative of possible solutions for retinotopic mapping. Our results confirm the robustness of the activation induced by phase-encoded designs for low-level areas identification and the consistency between data projection in most cases using one chain or the other. Beyond V3, delineation requires a careful mastering of the phase encoding technique, the introduction of additional knowledge or hypothesis as *a priori* information (like V4 having an hemifield rather than quarterfield representation Brewer et al., 2005) to obtain accurate and reliable maps for all subjects. Our results also indicate that the procedure to assign to individual retinotopic map the cortical activation following a cognitive experiment is not free of error. Consequently, the interpretation of cognitive activation projection onto fMRI retinotopic maps should be done cautiously. A preliminary version of this work has been previously presented in abstract form (Bordier, Hupé, Dojat, Human Brain Mapping Conference, Barcelona (Sp), 2010).

## 2. Materials and methods

### 2.1. Subjects

Ten healthy subjects (24–56 yrs, mean age 37±11, 3 males) with normal color vision, controlled using Lanthony D15 test (Richmond Products), were examined. All subjects but one had never participated in fMRI experiments. All subjects gave informed consent to participate in the study. The local ethics committee (Institutional Review Board of Grenoble) authorized the experiments (CPP 06-CHUG-23, approval date: 10/01/2007).

### 2.2. Experiments

All subjects participated in two fMRI experiments. Experiment 1, retinotopic mapping, was aimed at localizing retinotopic visual areas. Experiment 2, color center mapping, was aimed at localizing the functional responses produced by the introduction of chromatic contrast. Visual stimuli were created with Matlab (Mathworks, Inc., Natick, MA) and controlled during the experiment by a PC, running Windows XP with customized software written in C++ and using the SDL library for a precise control of timing. The stimuli were presented to the subjects using a video-projector (Epson 7250 M, Epson Inc., Long Beach, CA). Stimuli were back-projected on a translucent screen positioned at the rear of the magnet. Subjects viewed this screen at a distance of 222 cm via a mirror fixed on the head coil. Spectral and luminance calibrations of the display were performed with a PR-650 SpectraScan Colorimeter (Photoresearch).

### 2.3. Experiment 1: retinotopic mapping

A ring slowly contracting or expanding about the fixation point mapped eccentricity in the visual field. Speed of contraction or expansion varied linearly with eccentricity. Thus, the activation wave on the cortical surface traveled at approximately constant speed (under the assumption of an exponential cortical magnification factor). When the ring reached maximum eccentricity (maximal diameter extend of 6°) it was replaced by a new one at minimum eccentricity (set at 0.2°), and vice versa. Polar angle in the visual field was mapped by two wedges separated by a phase lag of 180° and rotating slowly at constant speed about the fixation point. Period of both eccentricity and polar angle stimulation was 32 s. Stimulus parameters were the same as described in Warnking et al. (2002): rings and wedges consisted of a black and white radial checkerboard flickering at a frequency of 4 Hz. Aspect ratio of the checks was kept constant by scaling their height linearly with eccentricity. The rationale underlying our choice of stimulation parameters may be found in Warnking et al. (2002). Successive stimulus images were presented at a frequency of 4 Hz, inducing the perception of a smoothly varying stimulus. Stimuli were started concomitantly with dummy MR excitations about 10 s prior to effective MR data acquisition in order to enable immediate response detection. To cancel out the effects of the hemodynamic delay, fMRI responses are compared to stimuli that cover the visual field in opposite direction clockwise and counter-clockwise for polar angle encoded stimuli, and expanded annuli and contracting annuli for the eccentricity encoded stimuli (Warnking et al., 2002). We acquired four retinotopic functional scans, one for each of the directions of motion of the rings and wedges.

### 2.4. Experiment 2: color center mapping

For the experiment three different types of events E0, E1, E2 (24 of each type) were programmed, in a pseudo-random fashion, at 2.5 s intervals. We used an experimental design with a constant occurrence probability for each type of event. The sequence of events was designed in order to optimize the efficiencies of the estimation both of the main and of the differential effects (Friston et al., 1999). Each event consisted in the presentation of an image during 1 s and inter-stimulus interval was 1.5 s. Since *TR* = 2 s, the occurrence of each event was desynchronized from the MR acquisition image.

The stimulus was a 6° by 8° rectangular field displayed on a gray background, Bg (luminance = 400 cd/m2; chromaticity: Judd CIE xy = 0.31, 0.38). The rectangular field was divided into 23 rectangles of various sizes. The rectangles were either assigned random chromaticities and luminance (E1, “chromatic event”) or random luminance (E2, “achromatic event”). The only constraint on the choice of chromaticities and luminances for each set was that the mean luminance of the rectangular field was equal to the luminance of the background Bg. E0 was a “null-event” for which the image presented was solely a fixation cross. During the “null-event” E0 and the inter-stimuli intervals a black centered fixation cross was presented on a uniform gray screen equal to the Bg.

### 2.5. Attentional task

The subjects were instructed to focus on the central fixation cross while paying attention to the whole stimulus. Eye movements were monitored (ASL EyeTraker 6000) over the course of the experiments. In order to maintain and control their own attention during the retinotopic mapping experiment, the observers had to press a button each time the very small (just visible) fixation cross, displayed centrally, briefly changed color or shape. During the color mapping experiment, they had to press a button each time a target (another cross) appeared briefly at a random position on the stimulus.

### 2.6. MR data acquisition

Images were acquired on a Bruker 3T Medspec S300 system whole body scanner (Grenoble MRI facility IRMaGE). A transmit-receive quadrature birdcage headcoil (Bruker) was used. Structural, functional, and static magnetic field (for B0 field inhomogeneity correction) data were acquired in a single scanning session. Static field homogeneity was optimized prior to acquiring the structural data, using the routine first-order shimming procedure. High-resolution structural images were acquired using a T1-weighted 3D MP-RAGE sequence optimized based on Deichmann et al. (2000). For each subject 176 sagittal partitions were acquired in two segments with an image matrix of 256 x 112 (read x phase). Further imaging sequence parameters were: TR/TE/TI: 16/4.96/903 ms, excitation pulse angle: 8°, acquisition matrix: 256 x 224 x 176 (CC, AP, LR), fast phase encoding direction: AP (112 steps per RAGE train, 2 segments), slow phase encoding direction: LR, isotropic nominal resolution: 1 mm, BW = 130 Hz/Px, readout direction: CC, number of averages: 1 and total measurement time: 14 min 40.

Functional data acquired during retinotopic and color stimulus presentation were obtained using a 2D, gradient-recalled echo (GRE), multi-slice, EPI MR sequence with the following parameters: TR/TE: 2000/30 ms, excitation pulse angle: 77°, acquisition matrix: 72 x 64 (AP, LR), isotropic nominal resolution: 3 mm, 30 adjacent contiguous slices, thickness 3 mm. Axial slices were angulated about the left-right axis to be approximately parallel to the calcarine sulcus. One of the central slices of the volume was positioned to contain as much of the calcarine sulcus as possible. Acquisition time per functional run was 7 min 50, allowing the acquisition of 235 volumes.

Images representing signal phase delays due to off-resonance (field inhomogeneity) effects were derived from two 3D GRE MR sequences differing in the echo time only (5.4 and 14.5 ms, respectively) and with the following common parameters: TR: 25 ms, acquisition matrix: 64 x 256 x 64 (LR,AP,CC), nominal resolution: 4 x 1 x 4 mm^3^. Slice orientation was set identical to the functional images.

These data were part of a functional study investigating which color sensitive areas are specially involved with colors induced by synesthesia (Hupé et al., 2012b).

### 2.7. Data pre-processing steps

Assigning functional responses to a surface model of the cortex is greatly sensitive to geometric distortions of the 3D functional data due to static field inhomogeneity. Geometric distortions, if not corrected, impact the quality of the retinotopic maps obtained (Vasseur et al., 2010). The inhomogeneity of the static magnetic field was taken into account during functional image preprocessing. Using the phase information in the GRE images acquired at different echo-times, a magnetic field map was first calculated (Cusack and Papadakis, 2002; Hutton et al., 2002). This was done with the SPM8 Fieldmap Toolbox. The magnetic field map was further used to compute a voxel displacement map and then to correct all the functional images for the geometric distortions and to realign them with respect to the first one of the series. The conjoint field correction and realignment procedure was realized using the SPM8 Unwarp toolbox. In a final step, all EPI data sets were aligned with the structural data set using the SPM8 rigid body coregistration procedure.

Spontaneous blinks during fMRI generate strong BOLD activation in the visual cortex and this activation strength is comparable to that evoked by visual stimulation (Hupé et al., 2012a). We analyzed the oculomotor signal using a custom interactive software developed in Matlab (Hupé et al., 2009) and using the basic data structure of ILAB software (Gitelman, 2002). Based on our expertise and the use of our interactive software, we properly identified the set of spontaneous blinks with a semi-automated procedure. For Experiment 2, for each run and subject, we computed a predictor based on eye blinks (blinks predictor). Eventually, we introduced the spontaneous blink predictor as covariate in our design matrix to remove corresponding blink activation.

### 2.8. Data processing

#### 2.8.1. Structural data

Using a distributed Markovian method (Scherrer et al., 2009), the structural image was segmented into three tissue types: cerebro-spinal fluid, white matter and gray matter.

#### 2.8.2. Volume-based analysis of color center data

We used the data of the color localizer sequence to identify hot spots of maximum color sensitivity in each subject. The logic and the precise procedure are detailed in the supplemental information of Hupé et al. (2012b). We used the ROIs defined for that study. Briefly, for each subject we used a conjunction contrast: we considered voxels as active if they responded more to both colored and achromatic Mondrian compared to the fixation point, and more to colored than achromatic Mondrians. We added a predictor based on eye blinks as a non-interest factor. For each subject, we first set the threshold at the 0.05 FDR level (False Discovery Rate), which value ranged between *t* = 3.37 and *t* = 5.21 (average *t* = 4.31). Then we increased the threshold in order to capture a larger number of active clusters within the ventral cortex (color centers), in or anterior to V4 (threshold values ranged between *t* = 2 and *t* = 3.9, average *t* = 3.18). We verified that the average response to chromatic Mondrians was above the average response to achromatic Mondrian (*t*-values in [2.76 6.34], average *t* = 4.59). These computations were performed in BV, in volume space. ROI volumes were exported to be used with BALC.

In order to obtain retinotopic maps, data were processed by two experts (CB and JMH), each skilled respectively in the use of Brain-A-La-Carte (BALC) and BrainVoyager (BV). Each expert analyzed the data with the tool he/she mastered. We briefly describe below the two chains; details can be respectively found in Warnking et al. (2002) and Goebel et al. (2006).

#### 2.8.3. Processing with BALC

First, the segmented structural image and the realigned and unwarped EPI functional images are supplied to BALC.

#### 2.8.3.1. Model of the cortical surface

Starting with the segmented image, the interface between volumes labeled as white matter and cortical gray matter was extended to represent approximately the center of the cortical surface. Some manual editing was performed to correct for topological defects detected in the occipital lobes. Then, the cortical surface located in the occipital lobes was selected manually from the segmented volume, for each hemisphere. The anterior and posterior limits were positioned respectively anterior and tangentially to the parieto-occipital and posterior and tangentially to the back of the occipital lobe. Within the delimited region, a triangulated model of the approximate center of the cortical surface interface was then generated following an approach based on the marching cubes algorithm (Lorensen and Cline, 1987). Distance between adjacent nodes of the triangulation was typically 1 mm. Data were then analyzed on the folded, triangulated surface model. A flattened representation of the surface model was eventually generated for visualization purpose and used for automatic tracing of visual area borders. Due to the position of the manually selected region to unfold, activation located in regions anterior to the parieto-occipital sulcus would not project onto the unfolded cortical surface. We could not guarantee that color functional activation observed in the volume would be systematically projected onto the flat map.

#### 2.8.3.2. Retinotopic map generation

The EPI datasets were analyzed voxel-wise by complex valued Fourier transformation, to determine amplitude and phase of the signals at the stimulation frequency (see details in Warnking et al. (2002)). The results of the volume-based analysis of the (four) retinotopic experiments were stored, for each visual coordinate, as a pair of parametric data volumes containing respectively phase and signal-to-noise ratio at the stimulation frequency. For generating retinotopic maps, phases were assigned to the cortical surface model as described in Warnking et al. (2002). The phase at each node of the surface model was estimated as a linear combination of the phases stored in a selection of voxels from the parametric data volume. Voxels from the parametric data volume that were retained in this selection had their center at most 3 mm away from the closest node of the surface model. Then, only voxels showing functional response with signal-to-noise ratio greater than two were selected (Warnking et al., 2002).

Following assignment of phase information to the surface model, retinotopic visual areas were automatically labeled and delineated on that model. This involved performing the following successive steps: (1). Application of a 2.5 mm Gaussian filter; (2). Calculation of a visual field sign map (VFS, Sereno et al. (1994)) that refers to the orientation of the representation of the visual field on the cortical surface. The orientation changes each time a border between area is crossed. For VFS determination, it is convenient to compute the Jacobian of the visual field representation onto the surface i.e., the ratio of an oriented surface measured in the visual field with respect to the same area measured on the cortex (VFR: Visual Field Ratio); (3). Identification of the low order visual areas as the set of voxels showing a VFR and a signal-to-noise ratio of the smoothed eccentricity and polar angle phase maps beyond preset thresholds (*SNR* > 3); (4). Selection of V1 using the hypotheses that V1 is the largest area and that the visual field sign changes between adjacent areas; and (5). Automatic identification of the borders among retinotopic visual areas as the contour lines of zero VFR. Delineation of the visual areas from the VFR map was entirely performed in the two-dimensional Cartesian space of the flattened surface representation. In some cases, based on a visual inspection of the polar angle map, some manual editing may be introduced to refine the visual areas borders, especially those to V3. The area borders are grossly parallel to direction of the calcarine sulcus. To bound the areas in the direction of the calcarine sulcus and define a surface for each area, we considered only the part between 0.2° and 3° of eccentricity that corresponds to the size of the stimulus for each hemisphere.

#### 2.8.3.3. Assigment to the cortical surface of color center mapping data

Similarly to retinotopic data, voxels from activated clusters were then assigned to the closest node of the cortical surface.

#### 2.8.4. Processing with BV

First, we imported the segmented structural volume and all the realigned and unwarped EPI functional images preprocessed with SPM8 to BV software. We are grateful to Denis Fize at CerCo who assisted us with the challenging exportation of SPM data (Analyze format) into BV non conventional 3D space. We applied a low trend removal and a high pass temporal filter (2/cycle) to each functional dataset.

#### 2.8.4.1. Model of the cortical surface

Using the segmented structural image, we created flat maps of the whole cortex (and not only of the occipital cortex as done with BALC) using the default pipeline (see “Cortex segmentation” section in the Methods of Goebel et al., 2006), but skipping BV tissue segmentation since that was already completed. Processing steps included white matter dilation and smoothing of the borders of the segmented data, separation of the left from the right hemisphere and application of a “bridge removal” algorithm, which ensured that the mesh representations of each hemisphere constituted single surfaces (Kriegeskorte and Goebel, 2001). For each hemisphere, the borders were then tessellated to produce a reconstructed model of the cortical surface and smoothed using 3D morphing iterative procedures. The 3D coordinates of this folded mesh remained the same as the original structural volume, allowing projection of functional data onto the surface. This folded mesh was therefore retained as the “reference mesh.” It was then inflated (iterative morphing algorithm), cut and unfolded to create a flat representation of the surface. The BV algorithm automatically performed five cuts within the medial side of each inflated surface. To make sure that one of these cuts ran along the calcarine sulcus, we specified manually landmarks (by clicking with the mouse on the inflated surface) within the depth of the calcarine. The cutting procedure did not succeed if holes were still present in the inflated surface, which happened frequently, usually because of holes in the frontal region around the anterior commissure. In that case we had to restart the procedure and manually edit the gray-white matter border on the original segmented volume. Once the inflated surface was properly cut, it was projected on the two sides of a flat map. The medial region that projected onto one side was then unfolded to create a single surface (iterative morphing algorithm). Unfolding creates major geometric distortions, so a further iterative algorithm was applied to minimize distortions. Flat maps were not isomorphic but they possessed a link to the folded reference mesh so that functional data could be shown at the correct location on the flattened representations. Regions of interest defined on the surface could be back projected within the volume (which is useful to average the BOLD signal time course within retinotopic areas defined on the surface). The entire procedure involved several smoothing steps, and we noted that small circumvolutions of the gray-white matter border were sometimes “lost“, especially in the ventral regions. This means that a few voxels, including some gray matter signal in the volume, would become distanced from the reconstructed surface. In that case, their signal could not project on the surface. This was particularly clear for regions in or around area V4: we observed functional activations in this volume by colored stimuli that would not project onto the flat surface. Based on such observations, we identified for each subject these critical regions, expanded the gray matter (therefore modifying the segmented structural volume) to improve the 3D projection onto the surface in these regions and repeated the entire cortex segmentation / flat map procedure. This refinement was only partly successful; therefore we could not guarantee that all color functional activations observed in the volume would systematically project onto the flat map. Note that these projections of color activations onto the BV surface were not used for our quantitative comparison between the two chains.

#### 2.8.4.2. Retinotopic map generation

We averaged (using Matlab) the two wedges and rings recordings, one of them read backwards before averaging. This procedure is convenient for generating modulated sinusoidal signals within retinotopic areas and cancelling out phase errors caused by hemodynamic delays (Warnking et al., 2002). With BV we computed correlation analyses with 16 sinusoidal functions with different phases (phase difference equal 0.2 rad between two consecutive functions) to obtain power and phase maps for both eccentricity and polar mapping. Phase maps were thresholded at a correlation of 0.2 (*t* = 3.15, *p* = 0.001) and projected on the cortical flat maps. V1 was identified as running along the calcarine sulcus. Borders between visual areas (identified as phase reversals) were then drawn by hand (and not automatically and then manually refined as done with BALC) on the polar phase maps, with simultaneous visualization of the eccentricity map to insure that the borders ran perpendicularly to the eccentricity gradient. This was done either on the inflated surface or the flattened representation (the coordinates of the vertices are the same). The inflated surface allows an easier identification of the sulci, borders form parallel lines on the flattened surface. For every subject and each hemisphere, we observed a half-field representation both ventrally and dorsally after the third phase reversal. This easy landmark helped us to identify areas V4 in the ventral cortex and V3a in the dorsal cortex in every subject. Dorsally and next to V3, other retinotopic areas (V3b, LO1, LO2) have been identified in several but not all subjects unambiguously. We observed areas VO1 and VO2 (Brewer et al., 2005) ventrally in a few subjects. Similarly to BALC, we bounded the areas in the direction of the calcarine sulcus to define a surface for each area in considering only the part corresponding to the size of the stimulus. Once created on the surface, these areas were back-projected in the volume so they could be exported and processed with BALC software for evaluation purpose (see below).

### 2.9. Evaluation strategy

#### 2.9.1. Map consistency evaluation

BV allows the definition of regions of interest both in the volume and the surface. The choice of the reference space belongs to the user. With BALC it is not possible to back project in the volume the retinotopic areas defined on the surface. Then, to compare the two chains we considered an unique referential i.e., the flat maps obtained using BALC. The retinotopic areas defined in BV were back projected in the BV volume, exported to be read by BALC, and then projected onto the individual BALC surfaces for comparison. To quantitatively assess the consistency between visual areas, we computed an overlap score in the BALC referential using the following equation (Equation (1)):

(1) $$overlap\_score_{i}=100*\frac{BV\;Area_{i}\;\cap \;BALCArea_{i}}{BV\;Area_{i}\;\cup \;BALCArea_{i}}$$

**Figure 1:**
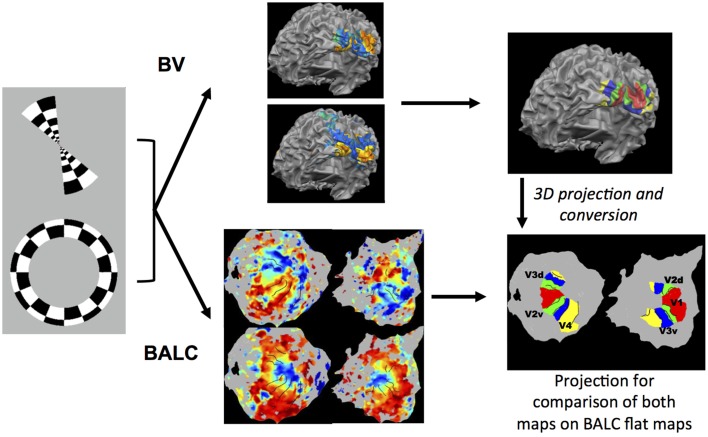
**Illustration of the methodology used**. Rings in expansion or contraction and wedges in rotation clockwise or anti-clockwise were used as visual stimulation. Corresponding functional images were first preprocessed with the identical SPM pipeline and then processed using two chains, BALC or BrainVoyager (BV). Structural images were segmented using an unique software (LOCUS) and only the surface reconstruction is software (BALC or BV) dependent. **Top**: with BV, starting from a segmented structural image, a 3D model of the cortical surface was reconstructed (here seen from the back and slightly below). Based on wedges (top) and rings (below) recordings, retinotopic areas were manually delineated and labeled on the surface. **Bottom:** using BALC, the region of each hemisphere posterior to the parieto-occipital sulcus was unfolded. Projection onto these surfaces of functional data corresponding to wedges (top) and rings (below) allowed the automatic delineation of the retinotopic areas. Lastly, the surface of retinotopic areas obtained using BV were back projected into the volume and then imported in BALC and projected onto reconstructed surfaces. Here the black lines are the borders obtained by BALC and the color surfaces were created with BV. Area surfaces obtained using the two chains may then be compared. Area labeling is the same for left and right unfolded hemispheres.

*BVArea_i_* and *BALCArea_i_* are the number of pixels contained in the considered area respectively for the delimited area BV and BALC. The numerator represents the common part (intersection) of the surfaces delineated by BV and BALC and the denominator the sum (union) of the surfaces. This metric was calculated for each subject, each area in each hemisphere. Figure 1 illustrates our methodology.

#### 2.9.2. Cognitive data projection

For Experiment 2, we expected differential activation to color vs. gray Mondrian stimuli in the majority of retinotopic areas with the stronger activation ventrally along the fusiform gyrus especially in V4. In Hupé et al. (2012b), the authors observe that the functional localization of such differential activation is highly subject dependent: any active cluster may be localized in and anterior to V4 in one or both hemispheres. Variations across individuals were also observed in Brewer et al. (2005). Here we tested whether such variability is dependent, at least in part, of the processing chain used. Using the two chains, we projected the same volume of individual active clusters onto BALC and BV and measured the overlap between projected clusters and the corresponding retinotopic area V4. We computed the overlap as:

(2) $$overlap\_score\_in\_V4\;=\;100*\frac{\begin{array}{c} the\;number\;of\;activated\\ voxels\;in\;V4 \end{array}}{\begin{array}{c} the\;total\;number\;of\\ activated\;voxels\;in\;the\\ ventral\;stream \end{array}}$$

For BV the computation was performed in the volume: V4 is the volumic ROI back projected from the surface V4 defined retinotopically. For BALC the computation was performed in the surface: activated voxels in V4 and the ventral stream were projected onto the surface model. We made sure that all the clusters used in the computations in the volume in BV did project to the BALC surface model. Contrary to the overlap score for map consistency that relies on the projection method used with BALC, which serves as the common referential, here the overlap is defined independently for the two chains. This allows a direct assessment of the consistency between two chains of the cognitive activation projection onto the individual retinotopic maps.

## 3. Results

### 3.1. Qualitative consistency between BALC and BV phase maps

**Figure 2:**
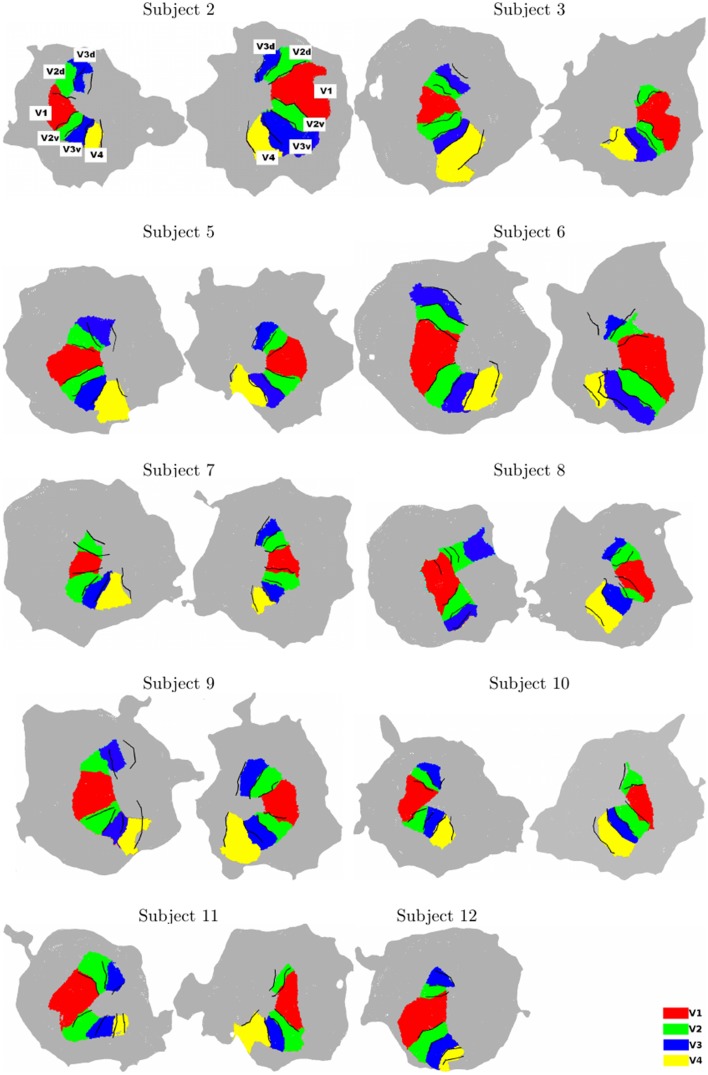
**Surface models for all subjects (Left: left hemisphere, Right: right hemisphere, for each subject)**. The surface models and borders corresponding to each visual area were computed using BALC (black lines). The surfaces of the visual areas (color patches) were computed using BrainVoyager and then projected on the BALC surface model. Some inconsistencies appear between the two methods in the dorsal part for two subjects (Subjects 8 and 9, left hemisphere) and BALC surface model failed for the right hemisphere of Subject 12. The visual area labels shown for Subject 2 are valid for all subjects.

For all subjects, using the two chains, we consistently identify ventral and dorsal parts of V1, V2, and V3. V4 represents a full hemifield in accordance with Bartels and Zeki (2000); Brewer et al. (2005); Cavusoglu et al. (2012); Wade et al. (2002); Wandell and Winawer (2011); Winawer et al. (2010). Note that V3A was not delineated using BALC due to the occipital cut made for the flattening but correctly manually defined using BV. Figure 2 shows the results obtained for all subjects. It reveals a good qualitative consistency between the maps obtained using the two chains. However, for 3 hemispheres (among 20) there was a clear inconsistency between the two chains. For Subject 8 (left hemisphere) the dorsal part of V2 and V3 obtained using BALC are respectively a part of V1 and a part of V2 dorsal when the delineation is based on BV. The signal was too noisy too delineate V4. Contrary, for Subject 9 (left hemisphere) the dorsal part of V2 and V3 as delineated using BV are respectively a part of V1 and V2 dorsal when the delineation relies on BALC. The dorsal part of V3 was not delineated using BALC. For the third incoherent case, the BALC reconstruction of a cortical model failed for the right hemisphere of Subject 12.

**Figure 3:**
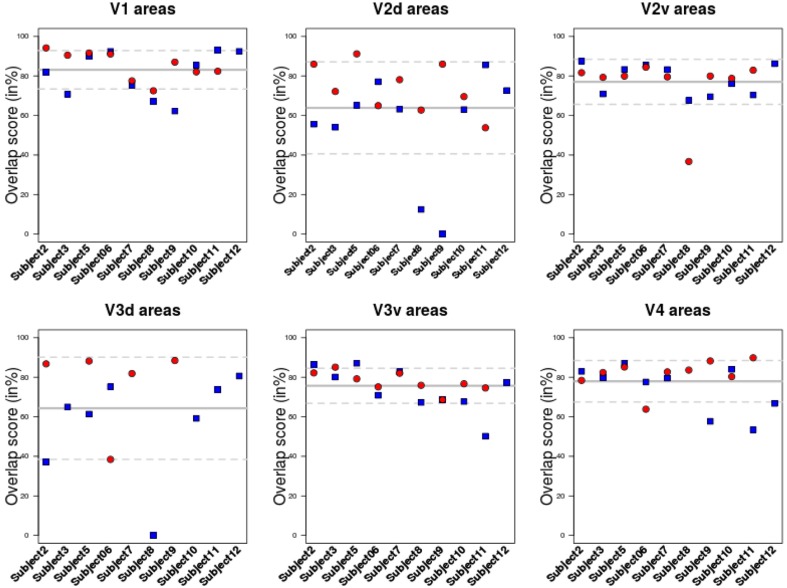
**Overlap score for all subjects in each area**. Each area was delineated either using BrainVoyager or using BALC. The comparison was performed in the BALC flat maps referential. Red: right hemisphere; Blue: left hemisphere. Solid gray line: mean value; dashed lines: the 95% Confidence Interval.

The overlap score between areas delineated using BALC and BV (see Equation 1) was computed for each subject, each area in each hemisphere. Table 1 reports the mean overlap in each area. Figure 3 shows overlap scores for all subjects in each area. V3 dorsal (V3d) was the most difficult to delineate automatically in BALC (effective delineation only for three subjects for both hemispheres), and V3A was almost never delineated with BALC due to the occipital cut made for the flattening.

**Table 1:** **Mean overlap between visual areas delineated using the two chains**.

	**All areas**	**V1**	**V2d**	**V2v**	V3d^*^	**V3v**	V4^**^
Mean overlap across subject (in% ± SD)	74.15 ± 16.9	83.11± 9.7	63.84 ± 23.2	77.02 ± 11.4	64.31± 25.8	75.7± 8.8	78± 10.5

Each mean overlap was computed on 19 delineated areas (on 20 hemispheres) but for

* (18) and

** (12)

The agreement between the maps obtained with two chains by two different users and processed manually and automatically is good. The percentage of overlap obtained from the two methods for the different visual areas (see Figure 3 and Table 1) is generally satisfactory, close to 90% (see Subject 12 and Subject 5 for V1) but can drop dramatically in dorsal stream (see Subject 8). This indicates that differences in image processing and manual correction of segmentation errors have an impact on final retinotopic maps, which remains acceptable for a majority of subjects.

### 3.2. Consistency between cognitive data projection

**Figure 4:**
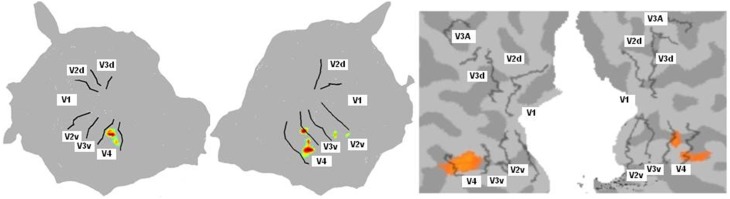
**Cognitive activation projection for Subject 10 on surface maps with the two chains**. Activations were obtained for the contrast chromatic vs. achromatic (Experiment 2). Only highly active clusters were projected onto the surface map. Left: Using BALC, Left: left hemisphere (95% of the projected pixels were included in V4); Right: right hemisphere (70% of the projected pixels were included in V4). Right: Using BV, Left: left hemisphere (76% overlap); Right: right hemisphere (73% overlap). In this example we show the overlap on the BV surface. The overlap score was however computed in the volume (the surface V4 area was back projected in the volume rather than projecting the activated voxels on the surface; see Materials and Methods).

For the color mapping experiment (Experiment 2), for the contrast Chromatic vs. Achromatic events, we expected the stronger activation ventrally along the fusiform gyrus especially in V4. The position and the number of active clusters are highly subject dependent (see Hupé et al., 2012b for a detailed discussion about color centers localization). Based on our data analysis (see Method section), each subject had between 1 and 7 active clusters. For most subjects, they lay in V4 and/or putative VO1/VO2. Figure 4 shows results obtained for one subject with BALC and BV respectively. As expected the regions known to be sensitive to color were the most active. It appears that both projections coherently show the same major activation.

**Figure 5:**
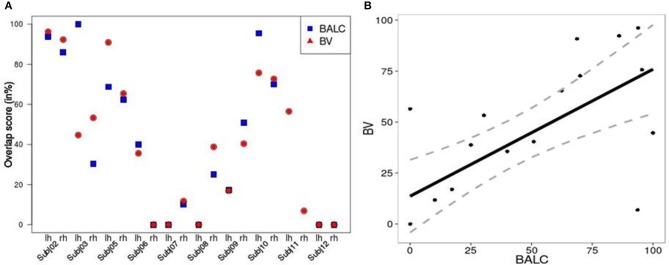
**(A)** Left: Overlap between area V4 and the activation by colored Mondrians (in blue using BALC and red using BV). **(B)** Right: Correlation between BALC and BV V4 area overlapping with the colored Mondrian contrast (dashed lines denote the 95% Confidence Interval).

We computed, for all subjects and each method, an overlap score (see Equation 2) between the number of voxels (or pixels) in the area V4 and the total number of activated voxels (or pixels) in the ventral stream. Figure 5A shows similar results for the two techniques except for 3 hemispheres (Subject 3 LH, Subject 11 LH and RH) among 20. For Subject 3, BALC found almost all activation due to color in left V4 compared to only 45% for BV. For Subject 11, BALC found no activation in left V4 while BV found 45%. The reverse was found for the right hemisphere. The graphic highlights that 3 subjects (Subject 5, Subject 7, and Subject 12) do not have any activation in area V4 when 2 others (Subject 2 and Subject 10) get more than 80% of there activation due to color in V4. For two subjects (Subject 8 LH and Subject 12 RH) V4 was not delineated using BALC. The degree of coherence between the two methods is illustrated by the correlation between BALC and BV shown in Figure 5B).

## 4. Discussion

Functional retinotopic mapping has become the current method for non-invasive delineation of visual cortical areas. It has largely contributed during the past twenty years to our knowledge of human visual cortex organization and our understanding of how information processing is distributed between visual areas (Wandell et al., 2007; Wandell and Winawer, 2011). Retinotopic maps are now routinely used in vision research and constitute a prerequisite when the precise assignation of functional activation to specific visual areas is an issue. Such maps provide also a means to define, based on visual area boundaries, matching regions of interest between subjects. In this paper we demonstrate that the maps obtained by skilled users are never fully identical. However, the agreement between the maps, around 80% for low-level areas, is probably sufficient for most applications. Note that all involved subjects but one were not familiar with fMRI experiments. We could expect more head movement and less sustained attention to the visual task compared to trained subjects. Nevertheless, the possible loss in signal to noise ratio did not alter the quality of the maps obtained. This also confirms the robustness of the activation induced by phase-encoded designs (Engel, 2012). Our results also indicate that assigning cognitive activations, following a specific experiment (here, color perception), to individual retinotopic maps is not free of errors. We provide measurements of this error, that may help for the cautious interpretation of cognitive activation projection onto fMRI retinotopic maps. On average, the magnitude of the misalignment of cognitive activations to the maps we delineated with the two methods was about 20% with much larger differences in a few subjects.

Functional retinotopic mapping involves several generic prone-to-error key steps: cortical surface reconstruction, 3D functional data projection to the surface and area borders delineation. Our study accurately documents the generated errors intrinsic to the procedure, implemented in two different ways and including a manual or automatic delineation of the area borders. Because other available software suites such as MrVista (Wandell et al., 2000), FreeSurfer (Fischl et al., 2001), or Caret (Van Essen et al., 2001) rely on the same generic steps, we may suppose that, similarly, differences would be observable in the majority of subjects. Our results demonstrate that despite differences in data processing, retinotopic maps are coherent between different chains. This indicates that individual functional retinotopic maps obtained in different centers with different procedures may be shared in freely available databases for the design of functional landmark-based probabilistic atlas of low level visual areas (Corouge et al., 2004) and anatomy to function correspondence studies (Abdollahi et al., 2014).

If delineation is strongly coherent for all subjects for V1, inconsistencies between the two methods appear in particular dorsally starting from V3 and ventrally around V4. When we succeeded with the delineation of 19 individual areas (over 20 hemispheres) for V1, V2v, V2d, and V3v, we obtained only 18 delineations for V3d and 12 for V4. As already indicated and due to the occipital cut made for the flattening, BALC results are poor for the dorsal part of V3 (see in Figure 3 the dispersion of the overlap score values for left and dorsal areas). However, when BrainVoyager seems to be able to delineate more areas, it requires many manual interventions and expert decisions which can lead to uncertainties.

Retinotopic acquisitions were not the main objective of the study: they have been recorded during a standard cognitive experiment with non familiar fMRI subjects and analyzed with two different methods which are not known to be the most used (and powerful). Although these conditions were not ideal, they correspond to the state of the art used in the majority of studies employing standard fMRI retinotopy mapping with cognitive experiments. Indeed, it is rather satisfactory that maps obtained by two different techniques are coherent because in numerous studies such maps are not detailed and exploited without any doubt or wariness. Commonly, activation induced by specific stimulation is then projected onto these cortex maps in order to explore which retinotopic areas are involved in information processing. Using a standard color center mapping experiment, we show (see Figures 4, 5) that there is good consistency between BrainVoyager and BALC for activation assignment to the cortical maps. Indeed, starting with data from this experience, we projected identical activations onto BrainVoyager surface and onto BALC surface. We then measured the overlap with the visual areas in each referential. By this way we have a direct measure of the difference between the two methods. Whether global coherence is noted, we observe differences in the assignment of activation to retinotopic areas. This indicates that we should be careful when interpreting the involvement, or absence of involvement, of a specific retinotopic area based on the projection of the corresponding cognitive activation onto individual fMRI retinotopic map. Incidentally, Figure 5B clearly indicates using the two methods that for fifty percent of the subjects, differential activation due to color does not entirely corresponds to the V4 area delineated based on the retinotopy property. This quantitatively confirms previous observations (Brewer et al., 2005; Hupé et al., 2012b) that color processing, highly variable between individuals, can not be attributed to a “specific color center“, located in V4, but is rather distributed in different visual areas.

Retinotopic maps are often ambiguous even to expert eyes. The boundaries can have irregular forms, some parts are identified with higher degree of certainty than others, and the exact location within the band of reversal phase is ambiguous (Kirson et al., 2008). An advantage of the automated method is that it does not require prior assumption about the visual field layout (Wandell et al., 2007). For the two methods, two skilled users, well familiar with retinotopic mapping and mastering the corresponding chains, processed data. Consequently, less consistency between maps might be achieved with less experienced users. Moreover, to limit the influence of data processing, the same SPM8-preprocessing steps and the same initial segmentation technique were used for gray matter delineation. Here we considered only retinotopic organization in V1 to V4 that is relatively easy to map. However, according to Dougherty et al. (2003); Winawer et al. (2010) the visual field center is generally critical to map. In order to improve precision in central fovea in limiting center surround suppression (Angelucci et al., 2002), we used a stimulus with an eccentricity of 3°, so with a maximal diameter extended of 6° from fixation, around two times lower compared to standard stimuli (e.g., Dougherty et al., 2003; Winawer et al., 2010). With the spatial resolution we used (3 mm isotropic), the mapping of central representation remained difficult. In spite of this methodological difficulty, a high agreement between the two approaches we used was observed in the current study. An extension to this work would be to consider high-level visual areas (as such VO1/VO2, LO1, LO2, etc ...). For this purpose, stimuli should be refined including a larger visual field to evoke stronger modulation in areas with large response fields, and a strong attentional task. Moreover, high quality in image acquisition and image processing chain are required in addition to a strong expertise in visual field representation for a precise delineation of higher-visual areas, dorsally such as V6 (Pitzalis et al., 2006) or in the intraparietal sulcus (Swisher et al., 2007), laterally in the lateral occipital cortex (Larsson and Heeger, 2006) and ventrally in the fusiform gyrus (Brewer et al., 2005; Arcaro et al., 2009).

In conclusion, the procedure to assign to individual retinotopic map the cortical activation following a cognitive experiment is not free of error. Here we provided quantitative measurements of this error, that may help for the cautious interpretation of cognitive activation projection onto fMRI retinotopic maps. On average, the magnitude of the error was about 20%, with much larger differences in a few subjects. More variability may even be expected by using different acquisition parameters and preprocessing chains.

### Conflict of interest statement

The authors declare that the research was conducted in the absence of any commercial or financial relationships that could be construed as a potential conflict of interest.
